# Reduced subgenual cingulate–dorsolateral prefrontal connectivity as an electrophysiological marker for depression

**DOI:** 10.1038/s41598-022-20274-9

**Published:** 2022-10-07

**Authors:** Lars Benschop, Gert Vanhollebeke, Jian Li, Richard M. Leahy, Marie-Anne Vanderhasselt, Chris Baeken

**Affiliations:** 1 Department of Psychiatry, Ghent University Hospital (UZ Gent), Corneel Heymanslaan 10, 9000 Ghent, Belgium; 2 Athinoula A. Martinos Center for Biomedical Imaging, Massachusetts General Hospital, Harvard Medical School, Charlestown, MA USA; 3 Department of Neurology, Center for Neurotechnology and Neurorecovery, Massachusetts General Hospital, Harvard Medical School, Boston, MA USA; 4 Ming Hsieh Department of Electrical and Computer Engineering, University of Southern California, Los Angeles, CA USA; 5 Department of Experimental Clinical and Health Psychology, Ghent University, Ghent, Belgium; 6 Department of Psychiatry, Free University of Brussels, Brussels, Belgium; 7 Department of Electrical Engineering, Eindhoven University of Technology, Eindhoven, The Netherlands

**Keywords:** Depression, Predictive markers, Psychology

## Abstract

Major Depressive Disorder (MDD) is a widespread mental illness that causes considerable suffering, and neuroimaging studies are trying to reduce this burden by developing biomarkers that can facilitate detection. Prior fMRI- and neurostimulation studies suggest that aberrant subgenual Anterior Cingulate (sgACC)—dorsolateral Prefrontal Cortex (DLPFC) functional connectivity is consistently present within MDD. Combining the need for reliable depression markers with the electroencephalogram’s (EEG) high clinical utility, we investigated whether aberrant EEG sgACC–DLPFC functional connectivity could serve as a marker for depression. Source-space Amplitude Envelope Correlations (AEC) of 20 MDD patients and 20 matched controls were contrasted using non-parametric permutation tests. In addition, extracted AEC values were used to (a) correlate with characteristics of depression and (b) train a Support Vector Machine (SVM) to determine sgACC–DLPFC connectivity’s discriminative power. FDR-thresholded statistical maps showed reduced sgACC–DLPFC AEC connectivity in MDD patients relative to controls. This diminished AEC connectivity is located in the beta-1 (13–17 Hz) band and is associated with patients’ lifetime number of depressive episodes. Using extracted sgACC–DLPFC AEC values, the SVM achieved a classification accuracy of 84.6% (80% sensitivity and 89.5% specificity) indicating that EEG sgACC–DLPFC connectivity has promise as a biomarker for MDD.

## Introduction

Major Depressive Disorder (MDD) is a severe, widespread and often recurring psychiatric illness that is primarily characterized by a loss of experiencing pleasure, persistent sad mood, sleep disturbances, changes in appetite and impaired concentration^1^. The lifetime prevalence of MDD is estimated around 20% and 30% for men and women respectively^2^. In addition, the societal and economic burden of MDD is considerable due to an increase in absenteeism, alcohol- and drug related issues, suicide attempts and general illness comorbidity, making MDD a leading cause of disability^3^. It is therefore imperative to focus research efforts on the development of reliable and practical biomarkers of MDD that can be applied in clinical settings.

In the past decade, research investigating neural correlates of MDD has shifted focus from identifying specific dysfunctional brain regions to examining intrinsic neural networks implicated in depression^4^. One of these networks is the cognitive control network (CCN) which comprises the dorsolateral prefrontal cortex (DLPFC), the anterior cingulate cortex (ACC) and the parietal cortex^5–7^. The CCN’s main function is to regulate cognition and behavior in the pursuit of internal goals by way of directing attention towards task relevant stimuli while simultaneously inhibiting task irrelevant stimuli^8,9^. In patients with depression the CCN’s connectivity is diminished, resulting in difficulties with both sustained attention and the downregulating of negative emotions^1,10,11^. There is mounting evidence showing the involvement of the CCN in regulating both positive and negative emotions through indirect top-down connections with limbic regions in which the subgenual ACC (sgACC) serves as a gatekeeper between the cognitive- and emotional network^12–17^. In short, the sgACC has dense connections with the amygdala and other regions of the limbic system^18–20^, constituting a brain network involved in emotional processing^4,21,22^. Moreover, the sgACC seems to project information from this affective network to the CCN’s frontal cortical regions^1,12,13^, resulting in top-down emotion regulation.

Abnormalities in the DLPFC and sgACC have been consistently found in MDD patients. Specifically, the sgACC seems hyperactive in depression and successful treatment leads to a normalization of this hyperactivity^23–25^. In contrast, the DLPFC has been found to be hypoactive in MDD patients^26–29^ and restoring DLPFC activity seems to elicit an antidepressant response^29,30^. As a result, both the left- and right DLPFC have become popular targets for noninvasive neurostimulation techniques such as Transcranial Magnetic Stimulation (TMS) in the treatment of severe depression^31–36^. Fox et al. posit that the left DLPFC and the sgACC are intrinsically anticorrelated during rest and that this anticorrelation is exacerbated in MDD^24^. Consistent with this notion, a neurostimulation study from Baeken and colleagues found stronger sgACC–DLPFC anticorrelations in responders before high frequency TMS treatment while observing a normalization in sgACC–DLPFC connectivity after remission^23^. Interestingly, applying low frequency TMS to the right DLPFC also seems to normalize sgACC hyperactivity^34^ and a meta-analysis that compared the clinical efficacy of both treatments found them equally effective^31^. Consistent with these neurostimulation findings, functional Magnetic Resonance Imaging (fMRI) studies reported reduced functional connectivity between the DLPFC and the dorsal ACC in late-life depression patients during an executive-control task^37^ and when at rest^38^. Taken together, aberrant sgACC–DLPFC connectivity seems to be a robust component of MDD, making it a promising candidate as a biomarker for depression.

A reliable biomarker should be an objective measurement of the physiologic or pathologic processes underlying a biological trait or illness^39^. Moreover, a biomarker that is being applied in diagnostics or treatment outcome should be relatively accessible for it to have any clinical relevance. Indeed, a biomarker has little clinical value if the operational costs are too high or the equipment necessary for measuring it is too scarce. For example, even though MDD biomarkers that are based on fMRI studies produce valuable information regarding the neurobiological underpinnings of the disease, they are seldom used in clinical practice. Since acquiring and operating MRI’s is expensive, hospitals will simply prioritize more urgent clinical matters than an MDD diagnosis. In contrast, the electroencephalogram (EEG) is a relatively affordable, time-efficient, and commonly available neuroimaging tool that is already routinely used in psychiatric and neurologic departments during a patient’s hospital admission^40^. Furthermore, EEG’s temporal resolution is superior when compared to most other neuroimaging techniques. For example, fMRI’s Blood Oxygenation level-dependent (BOLD) functional connectivity is based on the relatively slow hemodynamic response, restricting its temporal resolution to around 1 Hz. EEG can measure the brains electrical activity with a precision of milliseconds, resulting in a temporal resolution of more than 1000 Hz depending on the sampling rate capabilities of the amplifier. This allows researchers to estimate the functional connectivity of neural oscillations in real time over an extensive frequency range which can reveal unique electrophysiologic frequency signatures of neural processes.

One notable disadvantage of EEG is the low spatial precision which arises from the diffusion of the electrical signals caused by volume conduction of the skull^41^. In brief, electrical sources in the brain are projected on the scalp which are then measured by the EEG’s electrodes. However, volume conduction and the mixing of electrical signals introduces spatial artifacts, distorting scalp projections. For instance, a relatively small source localized in the occipital cortex could present as a large frontal projection^42^. These spatial artifacts are especially problematic when estimating functional connectivity since, diffused electrical signals measured by different electrodes could originate from the same neural source which would result in spurious connectivity values^43^. Fortunately, great strides have been made to minimize spatial artifacts stemming from volume conduction. Advancements in source estimation techniques such as the development of realistic head models^44,45^, high-density EEG (whole-head electrode coverage)^46^ and more reliable linear inverse solutions^47^ substantially improve the spatial accuracy of electrophysiological source models^48^. Consequently, numerous methods to estimate EEG resting state functional connectivity have been developed^49^. A study from Colclough and colleagues reports that out of 12 electromagnetic connectivity measures, amplitude envelope correlation (AEC) and partial correlation measures have the best intra-subject and between group consistency^50^; while another study found AEC to best mirror connectivity results obtained using fMRI^51^, making AEC an excellent measure of connectivity that can be compared across modalities.

While some EEG studies have looked at general spectral signatures of aberrant network functional connectivity in depression^52,53^, none have investigated whether disturbances in resting state sgACC–DLPFC functional connectivity could be utilized as a potential biomarker for MDD. Therefore, the aim of the current EEG study was twofold. Firstly, to replicate fMRI and neurostimulation studies that observed disturbances in connectivity between the DLPFC and the sgACC in depression and secondly, to evaluate whether this EEG sgACC–DLPFC functional connectivity has any reliable biomarker capabilities. The latter goal was attempted by training a support vector machine (SVM) on the estimated sgACC–DLPFC connectivity values and subsequently test the SVM performance to reliably distinguish individual MDD patients from healthy controls based on these connectivity values. Compared to other machine learning methods, SVM has some unique properties that are advantageous in the context of identifying psychiatric biomarkers such as the ability to analyze high-dimensional datasets with small sample sizes^54^. We chose to include a supervised machine learning approach, since accurately identifying MDD patients should be an essential attribute of any clinically relevant biomarker of depression.

## Methods

### Participants

The study sample is comprised of 40 participants including 20 MDD patients (13 females; mean age: 36.6, sd: 13.1) and 20 healthy controls (15 females; mean age: 41.25, sd: 14.64). The sample was originally collected for an ERP study performed by Vanderhasselt and colleagues in which they examined inhibitory control performance during MDD episodes^55^. Although the current study uses the EEG resting state of the same participants, it has no further relation with the ERP study beyond the concurrent collection of the data. The MDD patients were recruited from a psychiatric care facility where they were screened by a licensed psychiatrist using the Mini-International Neuropsychiatric Interview (MINI)^56^ and a structured clinical interview. Furthermore, all of the patients met the DSM-IV-TR diagnostic criteria of unipolar major depression and MDD severity was assessed with both the 17-item Hamilton Depression Rating Scale (HDRS)^57^ and the 21-item Beck Depression Inventory (BDI-II)^58^. Depression severity was repeatedly verified one week before- and at the time of testing, confirming the presence of MDD during data collection. Patient exclusion criteria included comorbid mood disorders with the exception of anxiety disorders, history of psychotic episodes or use of anti-psychotic medications, tricyclic anti-depressants and/or long-lasting benzodiazepines, a history of neurological conditions such as loss of consciousness for more than 5 min, head injuries, epilepsy, history of electroconvulsive therapy, past or present alcohol/substance abuse, and learning disorders. All patients were taking either Selective Serotonin Reuptake Inhibitors (SSRI’s) or Selective Noradrenalin Reuptake Inhibitors (SNRI’s) during data collection. Healthy controls were recruited through newspaper advertisements and had no history of depression or other psychiatric disorders. Additionally, all healthy controls were medication free, and all had a BDI-II score of < 14 and a HDRS score of < 7 during EEG data acquisition. All 40 participants received renumeration for participating and signed informed consent. Lastly, the medical ethics committee of the Ghent University Hospital approved of the study’s aim, methods, purposes, and the data collection was performed in accordance with the Ghent University Hospital ethics committee guidelines.

### EEG procedure and preprocessing

The EEG resting state data were acquired using a 128-channel Biosemi Active Two system (http://www.biosemi.com) within an electrically and acoustically shielded room. The EEG amplifier sampled the data at 512 Hz and employed an analogue filter with a bandwidth of 0.01–100 Hz. The data were referenced to the Common Mode Sense (CMS) active electrode and the Driven Right Leg (DRL) passive electrode. The EEG resting state was collected as 12-min segments (6 min eyes-closed and 6 min eyes-open, counterbalanced across subjects). Only eyes-closed segments were selected for analysis since the processing of visual scenes can have a confounding effect on the participants resting state data^59^.

A semi-automatic preprocessing pipeline was applied in MATLAB (version 2020b, The MathWorks, inc., Natick, MA) which utilized functions from the signal processing toolbox and the EEGLAB toolbox^60^. Digital offline filtering involved a 1 Hz high-pass and a 250 Hz low-pass filter. 50 Hz line noise was removed from the timeseries using the *cleanline* function which utilizes statistical thresholding to substract line noise estimations from the original signal^61^. Channels containing artifacts were identified using the *clean_artifacts* function using the following 3 parameters: (a) channel flatline lasting longer than five seconds, (b) channel noise exceeding four standard deviations relative to its own signal and (c) a correlation < 0.85 with its neighboring channels. General data cleaning was performed using the validated Artifact Subspace Reconstruction (ASR) method^62^. ASR decomposes the timeseries data into principal components and detects artifactual components by comparing specific components with components from the data’s cleanest segments. ASR then removes the artifactual components and reconstructs the timeseries with the remaining non-artifactual components. Before computing Independent Component Analysis (ICA), each subject’s EEG data was re-referenced to the average^63^ and a data rank correction was implemented. Following ICA decomposition, a Multiple Artifact Rejection Algorithm (MARA) was applied on the ICA components. MARA is a supervised machine learning algorithm that flags prevalent artifactual ICA components that represent eyeblink-, muscle-, noise- and electrocardiogram artifacts^64^. ICA components flagged by MARA were first visually inspected prior to removal. Lastly, omitted channels were interpolated using the spline interpolation method^65^ and both the preprocessed timeseries and its EEG frequency power spectra were visually inspected prior to data analysis.

### Preliminary analysis

A preliminary analysis was conducted in R (version 4.1.1) on the subjects’ demographic- and clinical questionnaire data. Chi-square and student t-tests were performed to examine if the study groups differed in age, sex, education, and depression severity.

### EEG source-space functional connectivity analysis

EEG functional connectivity was computed in source-space using the MATLAB toolbox Brainstorm^66^. The USCBrain atlas^67^ was selected as the shared brain anatomy template across participants. The USCBrain anatomy template (http://brainsuite.org/uscbrain-description/) is a high-resolution single-subject atlas that was created utilizing both anatomical- and functional data for cortex parcellation. Human connectome fMRI data^68^ from 40 subjects was used for the functional sub-parcellation which resulted in 65 regions of interest (ROIs) per hemisphere. Moreover, the Boundary Element Method was applied on the USCBrain anatomy template using OpenMEEG^44^ to produce a realistically shaped head model. This resulted in a head model that was identical for each subject. Individual sensor noise was estimated by calculating a noise covariance matrix on the resting state data of each participant. Only the diagonal elements of the noise covariance matrix were used as inputs to estimate sensor noise for the source localization model. Current density maps with unconstrained dipole orientations were generated using Minimum Norm^69^ as the source estimation method.

**Figure 1:**
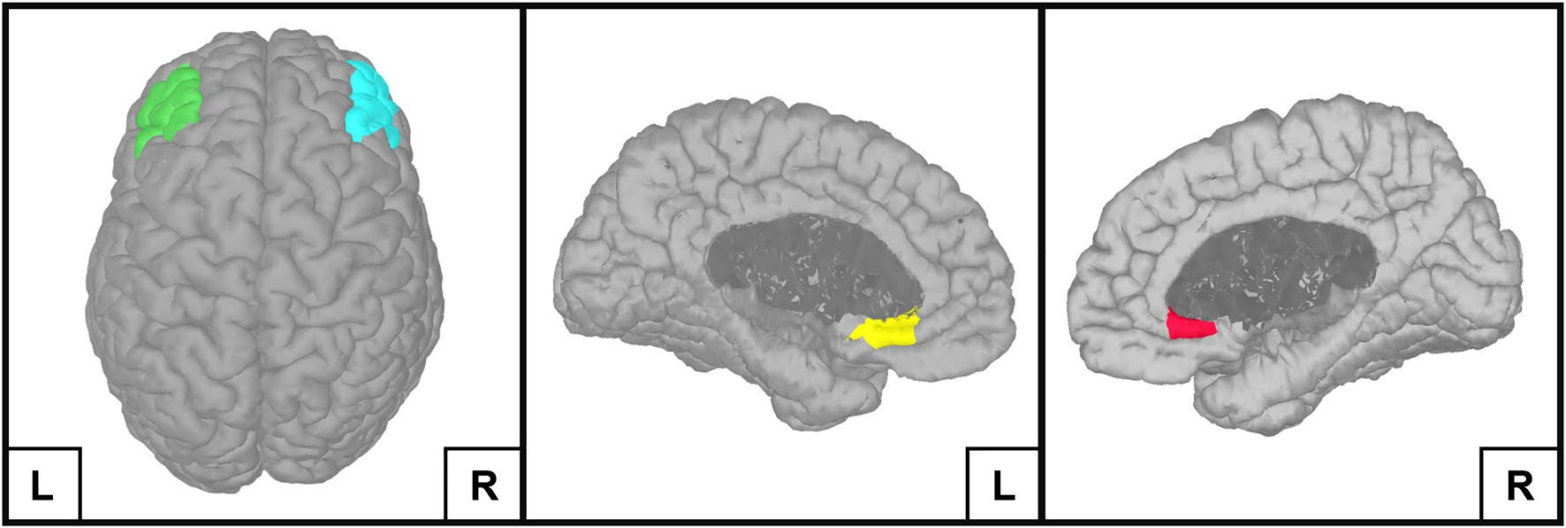
The four ROIs that were used in the source-space functional connectivity analysis performed in Brainstorm. The four ROIs include the left Dorsolateral Prefrontal Cortex (green), right Dorsolateral Prefrontal Cortex (turquoise), left subgenual Anterior Cingulate Cortex (yellow) and the right subgenual Anterior Cingulate Cortex (red).

**Figure 2:**
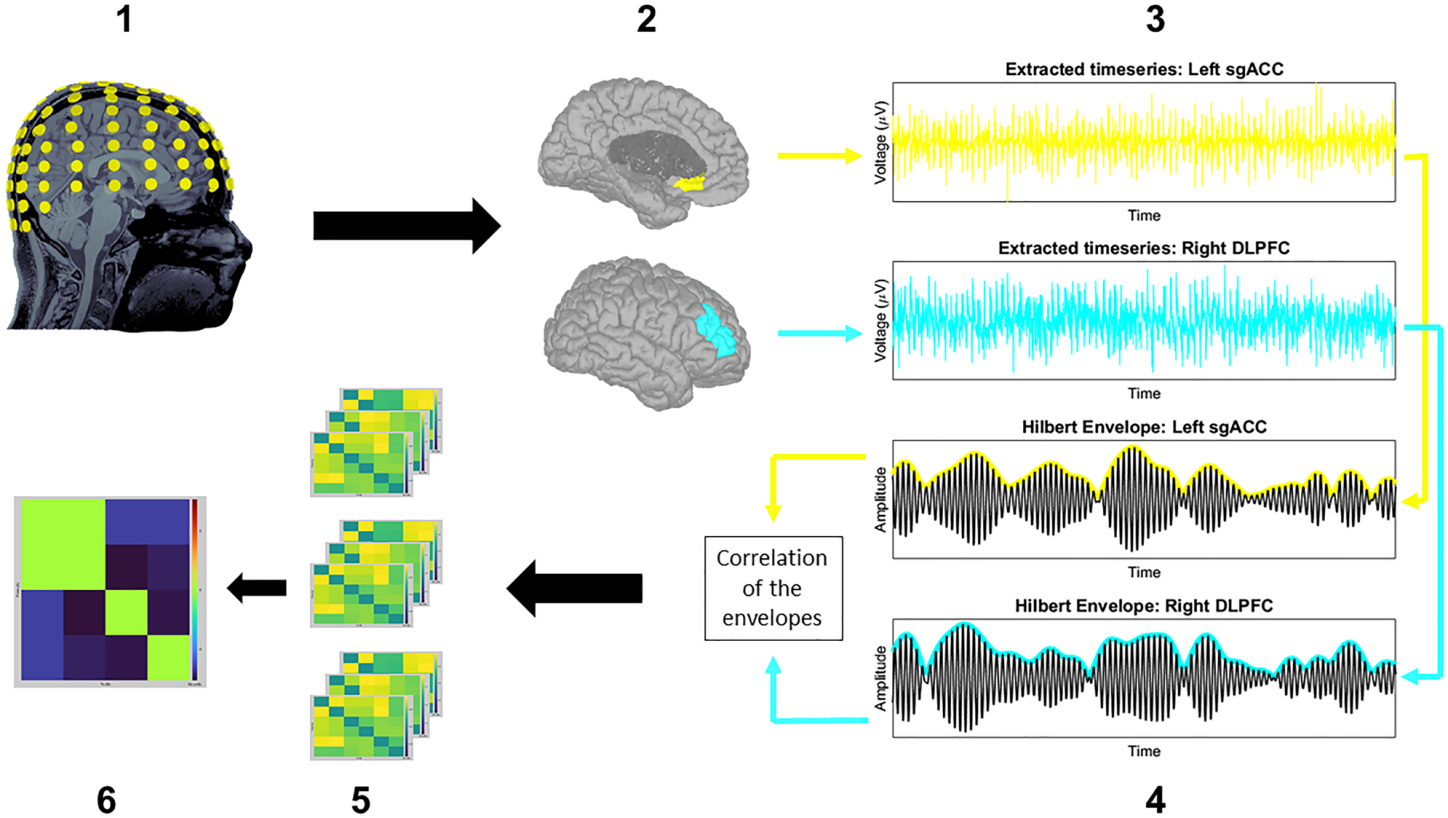
Estimating EEG source-space functional connectivity with the orthogonalized Amplitude Envelope Correlation method. The computation and statistical evaluation of orthogonalized Amplitude Envelope Correlations can be described in six steps: The EEG resting state timeseries data were transformed from sensor-space to source-space using the Minimum Norm source estimation method (**1**). Hypothesis driven regions of interest (ROIs) were selected from the USCBrain atlas (**2**) and their timeseries extracted (**3**) so that predefined band-pass filters together with an orthogonalization procedure could be applied. A Hilbert Transform was subsequently used to generate the power envelopes of these band-pass filtered signals (**4**). Correlations were then calculated between the power envelopes of different ROIs for each time sample. The average of these correlations was taken for each ROI pair which produced connectivity matrices for each frequency band of interest per subject (**5**). Non-parametric permutation tests were then performed, resulting in statistical maps that were thresholded using the false discovery rate (**6**).

Based on the growing neuroimaging literature that demonstrates a disunion between sgACC–DLPFC activity in MDD^23–25,29^ and the brain network model for depression^4^, the left/right DLPFC and left/right sgACC of the USCBrain atlas were chosen as ROIs for the source-space functional connectivity analysis (Fig. 1). Functional connectivity was estimated between these four ROIs using the AEC method described by Brooks and colleagues^51^ (Fig. 2). In brief, AEC values are calculated by applying a Hilbert transform to the ROI-extracted timeseries data, resulting in band-pass filtered analytical signals. The magnitude is taken from these signals and is used to compute power envelopes. Before these power envelopes are calculated, a symmetric orthogonalization procedure removes instantaneous signals that are shared between the different ROIs. This results in ROI-extracted power envelopes that have been corrected for spatial leakage artifacts which are a major source of spurious connectivity values^43^. Finally, linear correlations are calculated between the power envelopes of the different ROIs, resulting in a matrix that contains the averaged connectivity values for each prespecified frequency band. Since the frequency signatures of MDD are insufficiently understood, we opted to take a broadband approach in our analysis. Based on the findings of prior factor-analyses^70,71^, we investigated the following EEG frequency bands: theta (4–8 Hz), alpha (8–12 Hz), beta-1 (13–17 Hz), beta-2 (18–24 Hz), beta-3 (25–30 Hz) and gamma (30–100 Hz).

Differences between the two groups source-space functional connectivity values were statistically evaluated by performing two-tailed non-parametric permutation t-tests on the connectivity matrices. These resulting t-value maps were thresholded using the Benjamini and Hochberg’s false discovery rate (FDR) method^72^, allowing us to correct for multiple comparisons introduced by the multiple ROIs and frequency bands.

### Correlating EEG source-space functional connectivity values with MDD characteristics

The AEC values of the ROI pair with the strongest effect size were extracted and subsequently correlated with characteristics of depression, namely age of onset, lifetime number of episodes and duration of the current episode. The correlations were calculated in R using Monte Carlo permutations since AEC values are non-normally distributed. The association between MDD characteristics and AEC values was statistically evaluated using two-tailed, FDR-corrected tests of the Pearson’s correlation coefficient (*r*). In addition, a post-hoc analysis of covariance (ANCOVA) was used to examine if age, education level, sex and/or BDI-II scores had an influence on the association between connectivity values and MDD characteristics.

### Evaluating the Predictive power of functional connectivity data on MDD diagnosis using a SVM classifier

A SVM was used (the MATLAB *fitcsvm* function) to test the predictive power of left/right sgACC—left/right DLPFC connectivity on MDD diagnosis. The SVM classifier was trained using the extracted connectivity values of 4 ROI pairs (left/right sgACC—left/right DLPFC) to distinguishes MDD patients from healthy controls according to prespecified dichotomic labels. We used a Gaussian kernel and the Sequential Minimal Optimization solver with a box constraint (the regularization parameter) of 3. All other parameters were kept on their default values. Furthermore, the performance was evaluated using the ‘leave-one-out’ cross-validation method to avoid the overfitting issue.

## Results

### Participant demographic and clinical characteristics

As expected, MDD patients scored significantly higher on BDI-II and HAM-D scores when compared to healthy controls, demonstrating an ongoing episode of MDD at the time of testing. In contrast, the analysis revealed no significant differences between MDD patients and healthy controls regarding age, sex, and education level. The results and test-statistics of the participants’ demographic and clinical characteristics are outlined in Table 1.

**Table 1:** Participant demographic and clinical characteristics.

	HC (n = 20)	MDD (n = 20)	Test value	df	*p* value
**Demographics**					
Female, N (%)	15 (75)	13 (65)	χ^2^ = 0.12	1	0.73
Age in years, mean (SD)	41.25 (14.6)	36.6 (13.1)	t = 1.06	37.55	0.297
Education level, N (%)	HS = 4 (20)	HS = 8 (40)	χ^2^ = 2.93	2	0.231
	BA = 9 (45)	BA = 9 (45)	–	–	–
	MA = 7 (35)	MA = 3 (15)	–	–	–
**Symptomatology**					
BDI-II	1.45 (3.71)	33.45 (11.41)	t = − 11.37	20.22	< .001
HAM-D	0.2 (0.52)	27.56 (5.31)	t = − 21.78	17.3	< .001

Numerical data entries are in the form: mean (SD). Statistical evaluation was conducted with chi-square tests (χ^2^) and student t-tests (t).

*HC* healthy controls, *MDD* major depression disorder, *df* degrees of freedom, *HS* High School, *BA* Bachelor degree, *MA* Master degree, *BDI-II* Beck Depression Inventory II, *HAM-D* Hamilton Depression Rating Scale.

### EEG source-space AEC functional connectivity differences between MDD patients and healthy controls

**Figure 3:**
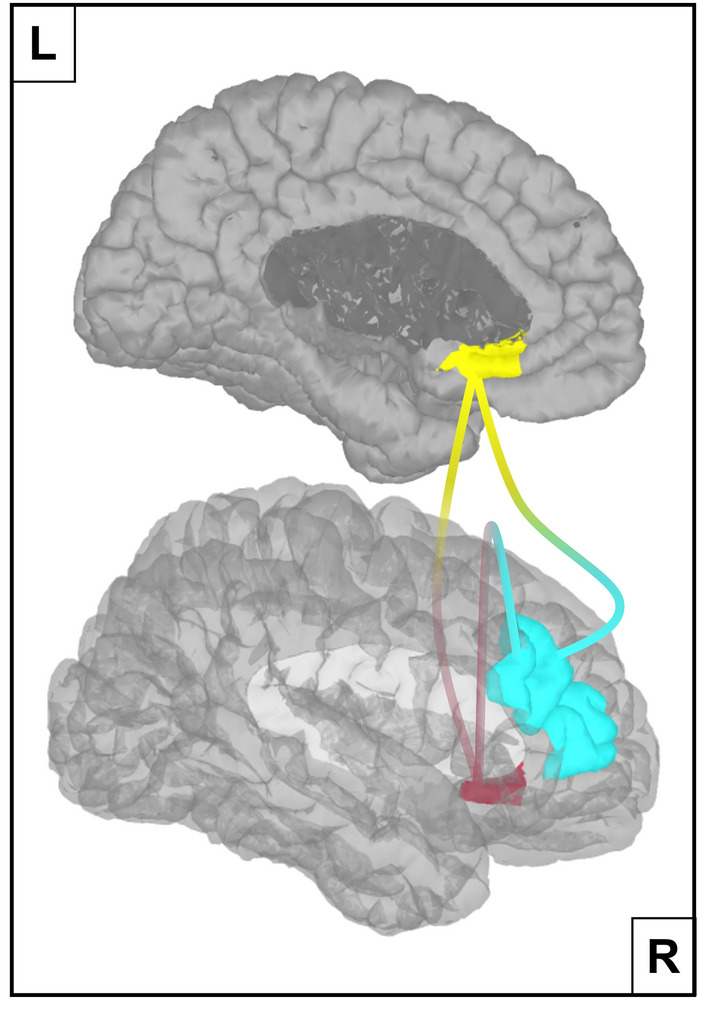
Diminished Amplitude Envelope Correlation connectivity in MDD patients. Depressed patients show reduced functional connectivity in the beta-1 (13–17 Hz) band for the following three ROI pairs: left sgACC (yellow)—right DLPFC (turquoise), right sgACC (red)—right DLPFC and left sgACC—right sgACC. This result was obtained after FDR thresholding. MDD: Major Depressive Disorder, sgACC: subgenual Anterior Cingulate Cortex, DLPCC: Dorsolateral Prefrontal Cortex, FDR: False Discovery Rate.

The two-tailed non-parametric permutation *t-*tests generated statistical maps that revealed significant differences in AEC functional connectivity between MDD patients and healthy controls within the beta-1 (13–17 Hz) and beta-2 (18–24 Hz) bands (*p* < 0.01, uncorrected for multiple comparisons). Both frequency bands showed diminished AEC functional connectivity for depressed patients and while this was limited to the left sgACC–right DLPFC ROI pair for the beta-2 band, all of the ROI pairs exhibited reduced connectivity within the beta-1 band. To control for the number of ROI pairs and frequency bands, an FDR threshold was applied (*q* < 0.05, adjusted *p* value = 0.0056) which yielded a thresholded connectivity map showing decreased AEC connectivity for MDD patients within the beta-1 band for the following three ROI pairs: left sgACC–right DLPFC, right sgACC–right DLPFC and left sgACC–right sgACC (Fig. 3).

### Association between AEC connectivity values and depression characteristics

**Figure 4:**
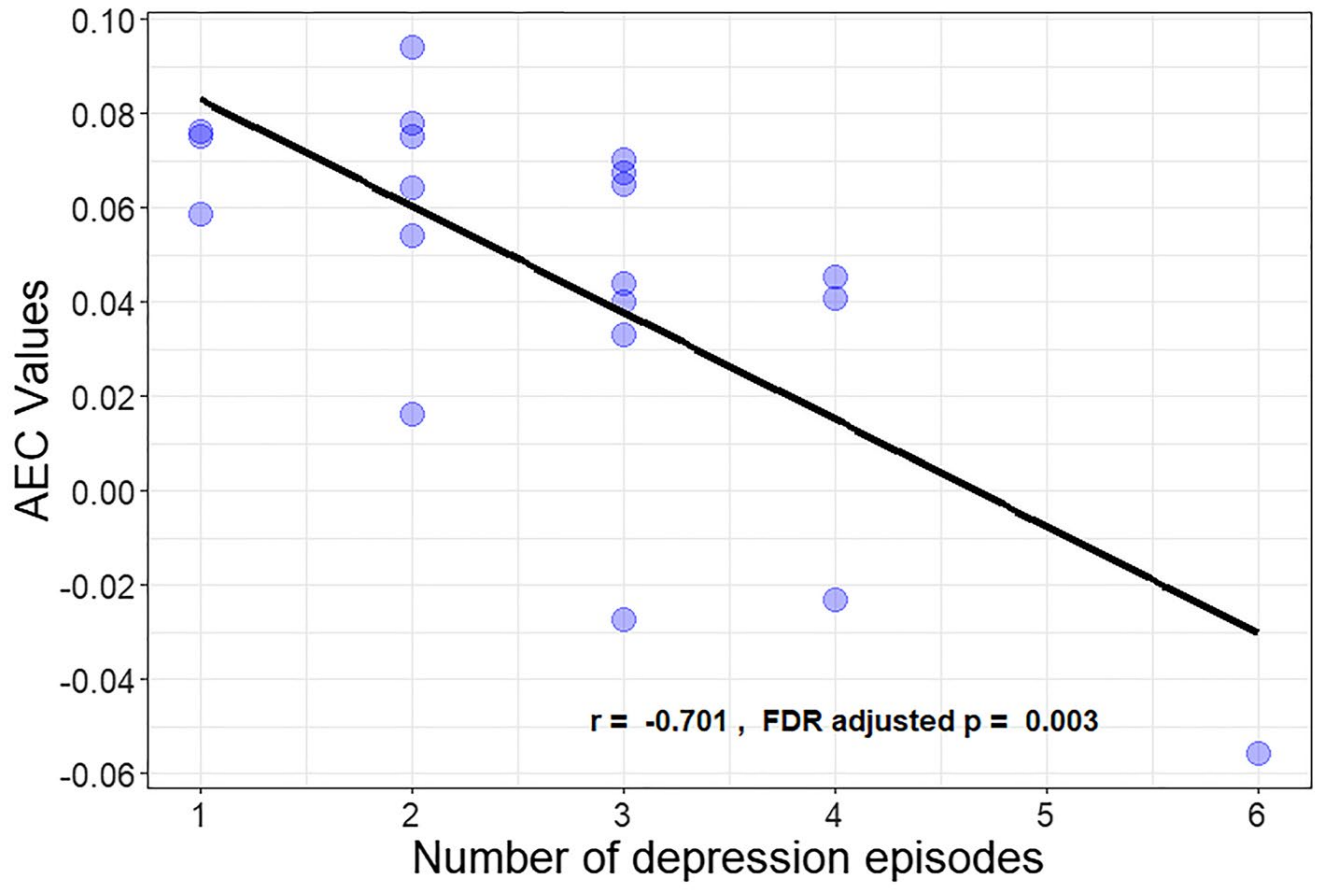
Scatterplot demonstrating the negative association between the number of depression episodes and sgACC–DLPFC Amplitude Envelope Correlation connectivity for depressed patients. Amplitude Envelope Correlation connectivity values from left sgACC–right DLPFC were correlated with the number of depression episodes a patient has had in his/her lifetime, using non-parametric Pearson correlation coefficients. sgACC: subgenual Anterior Cingulate Cortex, DLPCC: Dorsolateral Prefrontal Cortex.

Since the left sgACC—right DLPFC AEC values had the strongest effect size, they were extracted and subsequently correlated with MDD patients’ age of depression onset, lifetime number of episodes and duration of the current episode. The analysis revealed no significant correlation between patients age of depression onset and their extracted AEC values. However, a marginal negative association was found between current MDD duration and resting state functional connectivity (*r* = − 0.19, adjusted-*p* = 0.048, N = 20). More interestingly, reduced sgACC-DLPFC connectivity seems to be strongly correlated with the number of depressive episodes a patient has had in his/her lifetime (*r* = − 0.71, adjusted-*p* = 0.001, N = 20) (Fig. 4). Furthermore, this negative association remained significant after controlling for age, sex, education level and BDI-II scores (F(5,12) = 4.72, *p* = 0.014), suggesting that age and current MDD severity are not the driving factors between diminished sgACC–DLPFC connectivity and the total number of MDD episodes. This finding potentially signifies that reduced sgACC–DLPFC connectivity in the EEG resting state is a marker of a vulnerability to recurrent depressive episodes.

### Reduced sgACC–DLPFC connectivity as a diagnostic marker for MDD

Using the ‘leave-one-out’ cross validation method, the SVM classifier was able to successfully identify 80% of MDD patients (model sensitivity) employing the EEG resting state connectivity values between the left/right sgACC and the left/right DLPFC (4 features). Moreover, 89.5% of healthy controls (model specificity) were accurately identified, resulting in an overall model accuracy of 84.6%.

## Discussion

In order to minimize the extensive human- societal- and economic costs associated with depression, identifying usable markers of MDD will be an essential step toward that goal. Unfortunately, due to the infamous spatial issues associated with EEG in the past, research investigating EEG functional connectivity markers of depression is still in its infancy. To date, no EEG study has looked at aberrant sgACC–DLPFC connectivity within the resting state of MDD patients and thus our study addressed the question whether the EEG resting state contains disrupted sgACC–DLPFC connectivity within a sample of MDD patients and whether this aberrant connectivity has the potential to be utilized as a marker for depression.

Our findings revealed abnormal EEG resting state functional connectivity between the sgACC and the DLPFC in depressed patients. More concretely, depressive patients displayed diminished beta-1 (13–17 Hz) band AEC connectivity between the left/right sgACC and the right DLPFC when compared to healthy controls (Fig. 3). In addition, this reduced sgACC–DLPFC connectivity seems to be related to the total number of depressive episodes experienced during a patient’s lifetime (Fig. 4), even when age and current depression severity were taken into account. Lastly, sgACC–DLPFC connectivity within the EEG resting state seems to be a promising marker of depression when applying a SVM classifier, considering the relatively high model accuracy (i.e. 84.6%) based on only 4 features (left/right sgACC—left/right DLPFC) in this preliminary approach.

These results support the hypothesis of a negative association between DLPFC and sgACC activity in MDD patients when compared to healthy controls. Diminished sgACC–DLPFC connectivity in depression is likely to represent the inability of the CCN to inhibit a hyper-active limbic system which results in impaired top-down emotion regulation^4,10,11^. Furthermore, the DLPFC has been widely recognized to be an effective target site for noninvasive neurostimulation treatments with the aim to normalize DLPFC activity and to inhibit excessive sgACC reactivity which in turn reduces depression symptoms^73–75^. The DLPFC and sgACC seem to be inversely correlated in depression^24^ and successful TMS treatment normalizes this negative correlation^23^. Nevertheless, most studies report reduced functional connectivity between the left DLPFC and the sgACC while our results show diminished functional connectivity between the right DLPFC and the sgACC. Surprisingly, reducing the FDR threshold slightly (*q* = 0.06, adjusted *p*-value = 0.009) also reveals reduced connectivity between the left DLPFC and both left/right sgACC in the current study. It is possible that both the left- and right DLPFC have impaired connectivity with the sgACC in depressed patients, and this notion is supported not only by our own findings but by a meta-analysis that did not find a difference in clinical efficacy between TMS stimulation of either the left- or right DLPFC for treating depression^31^. In addition, Kito and colleagues reported reduced sgACC activity after successful low frequency TMS stimulation of the right DLPFC in treatment resistant depression patients^34^, demonstrating an overlap of therapeutic working mechanisms with high frequency TMS stimulation of the left DLPFC. Furthermore, the current data demonstrate that reduced EEG sgACC–DLPFC functional connectivity seems to be related to the total number of depressive episodes within a patient’s life. Remarkably, one of the few EEG studies that looked at disrupted network connectivity in depression found an association between the frequency of depressive episodes and hyperconnectivity between the Default Mode Network (DMN) and the CCN, also within the beta-1 band^53^. This between-network beta-1 band hyperconnectivity may reflect a weakened CCN being “hijacked” by an overactive DMN^76,77^, sharing an electrophysiologic signature similar to the CCN’s inability to regulate the sgACC hyperactivity. Although, this interpretation is compatible with our current finding, it is unable to address if aberrant EEG CCN connectivity reflects a biological vulnerability for a recurrent illness course or if recurrent depressive episodes increase the intensity of network abnormalities. Further support for an association between impaired cognitive control and the lifetime number of depression episodes can be found in a 2009 ERP study^78^. The authors observed an inverse correlation between the amplitude of a cognitive control-related ERP (N450) and the number of prior MDD episodes in a sample of remitted depression patients. These results not only corroborate the cumulative nature of cognitive control impairments in depression but also demonstrate that these deficits seem to persist, even when patients are in remission. This association between impaired cognitive control and recurrent depressive episodes is consistent with the kindling hypothesis of recurrent depression^79^. The kindling hypothesis states that psychosocial stressors become less relevant for depression onset with each subsequent depressive episode and that the onset of successive MDD episodes are increasingly independent of environmental influences and events. It is entirely plausible that impairments in cognitive control and difficulties regulating negative emotions increase the likelihood of future depression episodes through a reduction in an individual’s ability to cope with life events, big or small.

Some fMRI studies report divergent findings in which sgACC–DLPFC connectivity is enhanced in MDD patients. One study found a positive correlation between posterior sgACC–DLPFC connectivity and BDI-II scores in subjects with subclinical depression^80^, while Davey and colleagues observed increased pregenual ACC—left DLPFC connectivity in the fMRI resting state of depressed patients^81^. A plausible explanation for these inconsistent findings is that the DLPFC can become hyperactive in depression in an attempt to downregulate sgACC hyperactivity^24^. This hypothesis might seem inconsistent with our own observations but can potentially be explained by the differences in temporal resolution between EEG and fMRI. Since EEG measures neural activity directly, it is able to detect the instantaneous high frequency (> 4 Hz) discordant activity patterns between the sgACC and the DLPFC. In contrast, the slower and indirect BOLD signal of fMRI measures a low frequency (< 0.15 Hz) timeseries that could represent DLPFC activity that lags behind the hyperactive sgACC, resulting in increased connectivity values between the two regions. Similarly, a TMS-EEG study from Hadas and colleagues reported decreased effective connectivity between the left DLPFC and the sgACC after successful TMS treatment^82^. In addition, the reduction in left DLPFC–sgACC connectivity was associated with MDD symptoms improvement. This result seems to directly contradict our own observations of reduced DLPFC–sgACC connectivity within a sample of MDD patients. A possible reason for these divergent findings could result from methodological differences with respect to how connectivity was estimated. Our own study applied AEC on band-pass filtered signals to compute functional connectivity, whereas the study from Hadas and colleagues applied a form of effective connectivity on (broadband) current source density values (i.e. source localized voltage values). This explanation seems plausible, since other EEG resting state functional connectivity studies did observe increased beta-band connectivity between the DLPFC and the sgACC within MDD patients after either a successful TMS-^83^, or pharmacological treatment^52^, corroborating the current findings. In addition, Hadas and colleagues looked at DLPFC—sgACC connectivity changes within depression patients almost immediately after TMS stimulation (i.e. within a 500 ms interval), while our study examined EEG functional connectivity between depressed- and healthy participants at rest.

Lastly, sgACC–DLPFC AEC functional connectivity seems to have potential as a diagnostic marker for MDD within a machine learning framework. The model performance of 80% to accurately classify depression patients based on left/right sgACC—left/right DLPFC connectivity values is promising. Nevertheless, some considerations need to be kept in mind when interpreting machine learning classification results. The current study’s sample size is small and as a result overfitting can become a problem^84^. This is especially problematic in neuroimaging research that employs a large number of features^85,86^. In order to address this issue a SVM classifier was used which can be considered suitable when working with limited sample sizes. Additionally, the total amount of features was constrained through selecting hypothesized connectivity differences between patients and controls. One advantage of this approach is that it is less likely that model’s performance is based on irrelevant features specific to the current dataset. Another advantage of using a hypothesis driven feature selection approach is the increase in reliability and interpretability of the results. The disadvantage is that potentially valuable features (such as connectivity with other relevant regions) could have been excluded.

Future studies should therefore expand on these findings by attempting to replicate the current study in sizeable datasets. Besides increasing the validity of potential diagnostic markers, it would significantly improve the signal-to-noise ratio as well. This increase in signal power would allow researchers to reliably compute measures of dynamic functional connectivity such as estimating AEC values within predefined sliding time windows. These kind of EEG analyses can offer valuable insight into the temporal aspects of disrupted neural networks in MDD. Furthermore, an excellent signal-to-noise ratio would make it possible to develop certain spatial- and temporal filters that could drastically reduce the number of electrodes needed to reveal markers of depression, further boosting clinical applicability.

In conclusion, our results revealed diminished sgACC–DLPFC connectivity within a sample of MDD patients when compared to a sample of healthy controls. In addition, this reduced sgACC–DLPFC connectivity was associated with the total number of depression episodes a patient has experienced at the time of testing. The diagnostic power of this aberrant connectivity was evaluated using a SVM classifier which resulted in a model performance of 80% sensitivity, 89.5% specificity and 84.6% accuracy, suggesting that sgACC–DLPFC functional connectivity could be a potential diagnostic marker of depression.

## Data Availability

The data used in this manuscript are available from the corresponding author (Lars Benschop) on suitable request. The data is not publicly available due to the privacy and ethical restrictions regarding Ghent University Hospital’s patients privacy protection policy.
